# Supplementary material for: A cell fitness selection model for neuronal survival during development
Source: Nat Commun. 2019 Sep 12;10:4137. doi: 10.1038/s41467-019-12119-3 (PMC6742664; doi:10.1038/s41467-019-12119-3)
Supplement: Supplementary file 1 — Supplementary Information [file 41467_2019_12119_MOESM1_ESM.pdf]

SUPPLEMENTARY INFORMATION

A cell fitness selection model for neuronal survival during development

Wang et al.

Supplementary Figures 1-8
Supplementary Table 1
Supplementary References

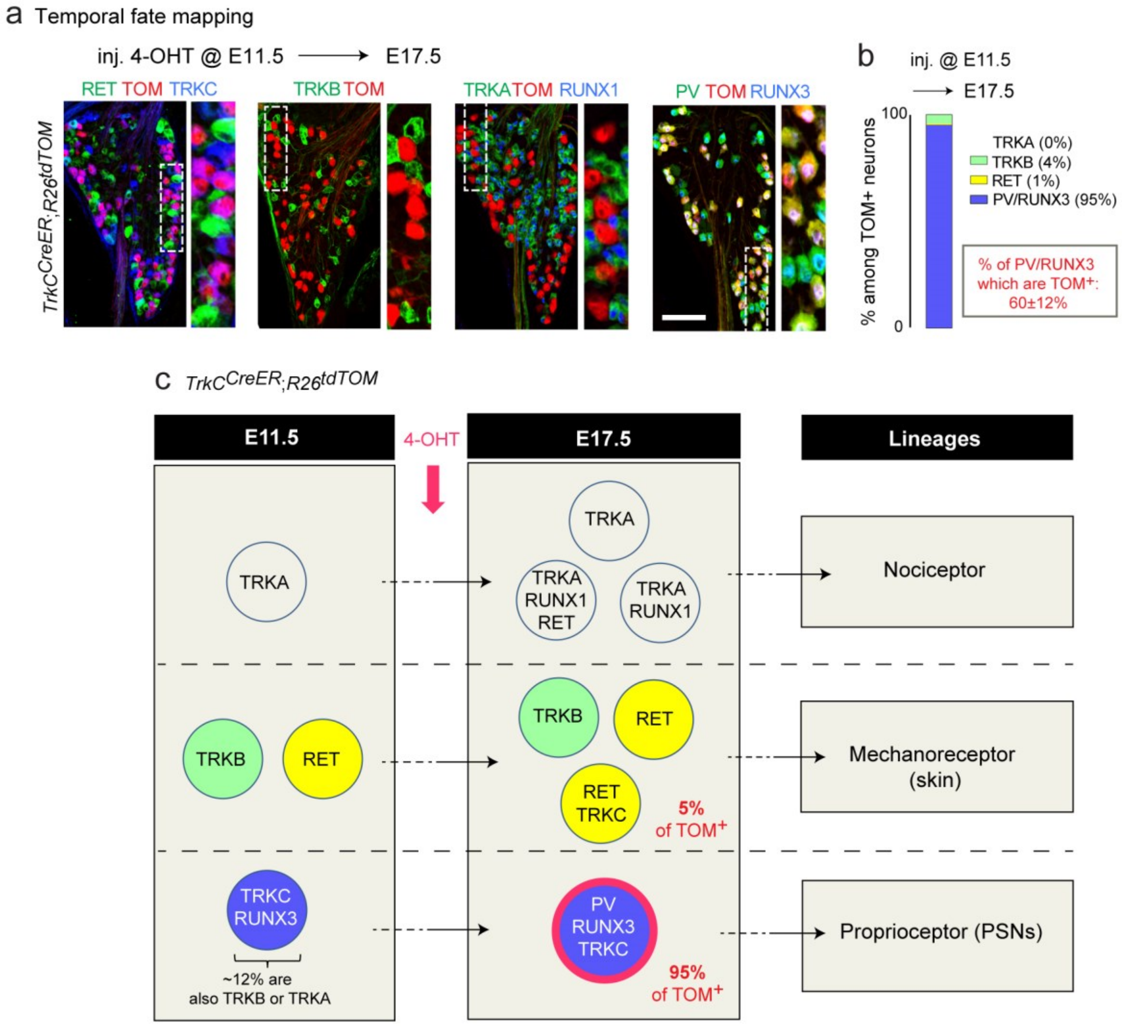

Supplementary Fig. 1. Temporal fate mapping of presumptive PSNs. a, Immunostaining on E17.5 DRG sections of *TrkC*^{CreER};R26^{tdTOM} mice after temporal fate mapping of TRKC positive neurons by 4-OHT induction (high dose, 0.08g/kg, injected at E11.5). Markers used reveal the different neuronal lineages at E17.5: mechanoreceptors (RET/TRKC, TRKB or RET populations), nociceptors (TRKA/RUNX1 or TRKA populations) and proprioceptors (PV/RUNX3 neurons) n=4. Scale bar: 100µm. **b**, Quantification of the percentage among the TOM$^+$ cells from (**a**) show predominant recombination within the PV$^+$/RUNX3$^+$ population of neurons (95%), identified as PSNs at E17.5[1,2]. Note that the recombination rate within the PV$^+$/RUNX3$^+$ PSNs was 60±12% (mean ± SD, n=4). Hence, the large majority of TRKC$^+$ neurons at E11.5 become PSNs. **c**, Scheme representing the lineage fate mapping of 4-OHT injection at E11.5 in a *TrkC*^{CreER};*R26*^{tdTOM} mouse line. 95% of the TOM$^+$ labelled neurons at E11.5 are within the PSNs lineage.

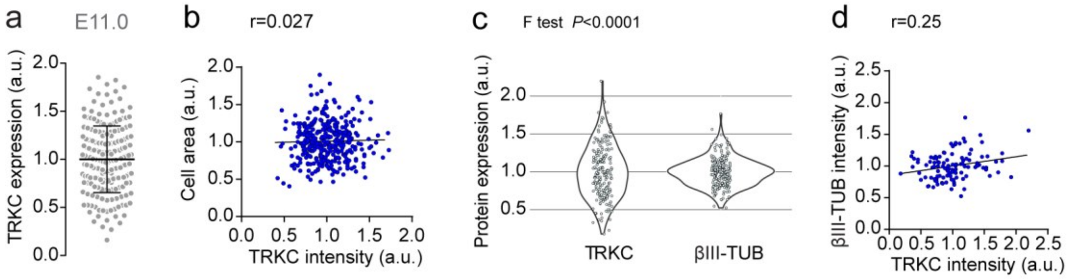

Supplementary Fig. 2. Heterogeneity in TRKC levels in PSNs before the cell death period. a, Distribution of TrkC levels in PSNs in E11.0 RUNX3^{+} DRG neurons, showing large heterogeneity already at this early stage. **b**, No correlation was observed between cell area and TRKC intensity (r=0.027). **c**, Distribution of TRKC and βIII-tubulin (B3-TUB) levels in PSNs in E11.5 DRG individual neurons (represented with SD; n=2 embryos, 79 neurons). Note that the F test compares the variances has a $P < 0.0001$ implying that the spread of distribution of βIII-tubulin and TRKC are significantly different. **d**, No significant correlation between βIII-tubulin and TRKC expression level (r=0.25).

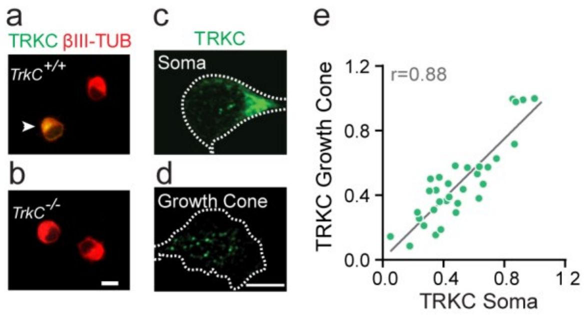

Supplementary Fig. 3. TRKC protein expression correlates between soma and growth cone. **a**,**b**, TRKC expression using TRKC antibody on neurons in DRG cultures from E11.5 WT or *TrkC* null mice. Note the positive staining in WT condition (yellow cell, arrowhead) (**a**). In contrast, not a single neuron was positive for TRKC staining in the *TrkC* null DRG cultures (see *TrkC* null condition) (**b**), confirming the specificity of the antibody used in our *in vitro* experiment in. **c**,**d**, TRKC expression in soma (**c**) and growth cone (**d**) of E11.5 DRG neurons cultured 6 hrs with NT3 (10 ng/ml). **e**, Correlation between the levels of TRKC expression in the soma and growth cone of individual PSNs grown in culture as in (C) and (D). Scale bars: 5μm.

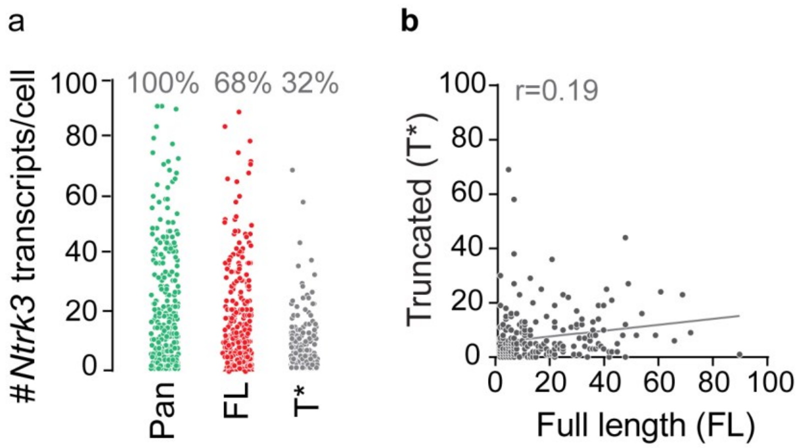

Supplementary Fig.4. Characterization of TRKC isoforms expression in E11.5 DRG neurons. a, Distribution of the number of pan *Ntrk3*, *Ntrk3* FL and *Ntrk3* T^* (truncated, extrapolated value Pan *Ntrk3* minus *Ntrk3* FL)[3] molecules in individual E11.5 DRG neurons by smFISH, normalized to the maximum (Pan *Ntrk3*, 100%). **b**, No correlation between the number of *Ntrk3* FL and *Ntrk3* T^* molecules in individual E11.5 DRG neurons from (**a**). Data show high cell-to-cell variable expression of the *Ntrk3* FL isoform while that of the truncated isoforms was comparatively lower and displayed more uniform expression amongst PSNs

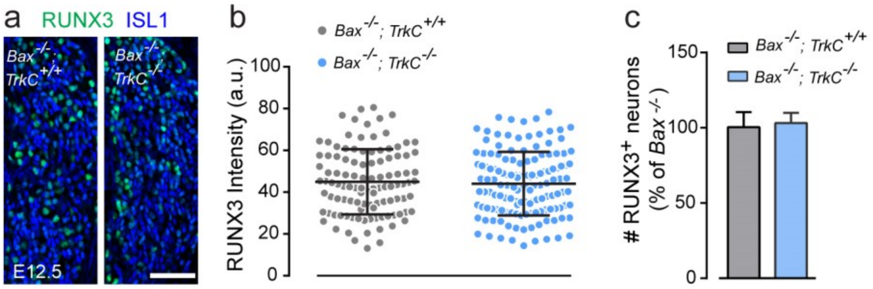

Supplementary Fig. 5. RUNX3 expression is independent of NT3-TRKC signaling. a, TRKC and ISL1 expression in E12.5 DRG sections from *Bax*$^{-/-}$ and *Bax*$^{-/-}$;*TrkC*$^{-/-}$ animals. Scale bar: 50µm. **b**, Quantification of TRKC levels in individual PSNs from E12.5 *Bax*$^{-/-}$ and *Bax*$^{-/-}$;*TrkC*$^{-/-}$ animals (n=3 embryos). **c**, Quantification of RUNX3^{+} neurons in E12.5 *Bax*$^{-/-}$ and *Bax*$^{-/-}$;*TrkC*$^{-/-}$ DRG sections (n=4, data are normalized to *Bax*$^{-/-}$ condition).

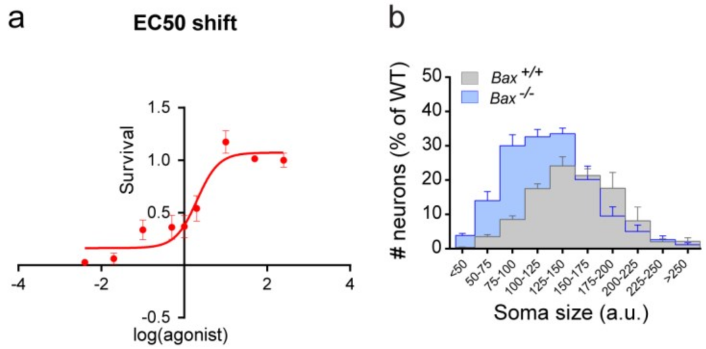

Supplementary Fig. 6. EC50 of NT3 for DRG neurons and PSNs soma size analysis in E13.5 *Bax* null mice. a, EC50 of NT3 for DRG neurons, concentration/survival curve of NT3 on E11.5 DRG neurons cultured for 48 hours. Data are normalized to maximum survival, and obtained (from samples composed of n=6-8 embryos DRG) from 4 different experiments. **b**, Quantification of soma size in PSNs from E13.5 $Bax^{+/+}$ and $Bax^{-/-}$ DRG (n=3).

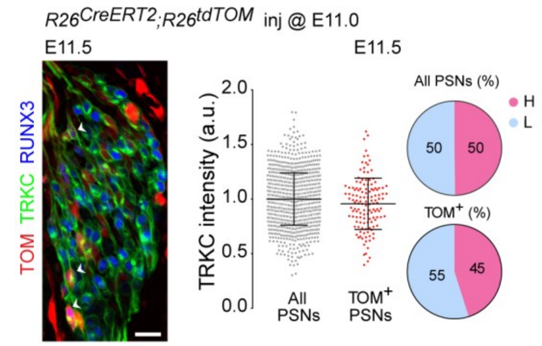

Supplementary Fig. 7. Genetic tracing of PSNs *in vivo*. Injection of $R26^{CreERT2}$;$R26^{tdTOM}$ embryos at E11.0 with a low dose of 4-OHT (0.032g/kg; n=4) showing recombination in few PSNs at E11.5. Graph and pie charts show that the TOM^{+} PSNs are represented in all TRKC intensity categories with the same proportion than for TOM^{-} PSNs (compare with Fig. 3c,d). Scale bar: 200μm.

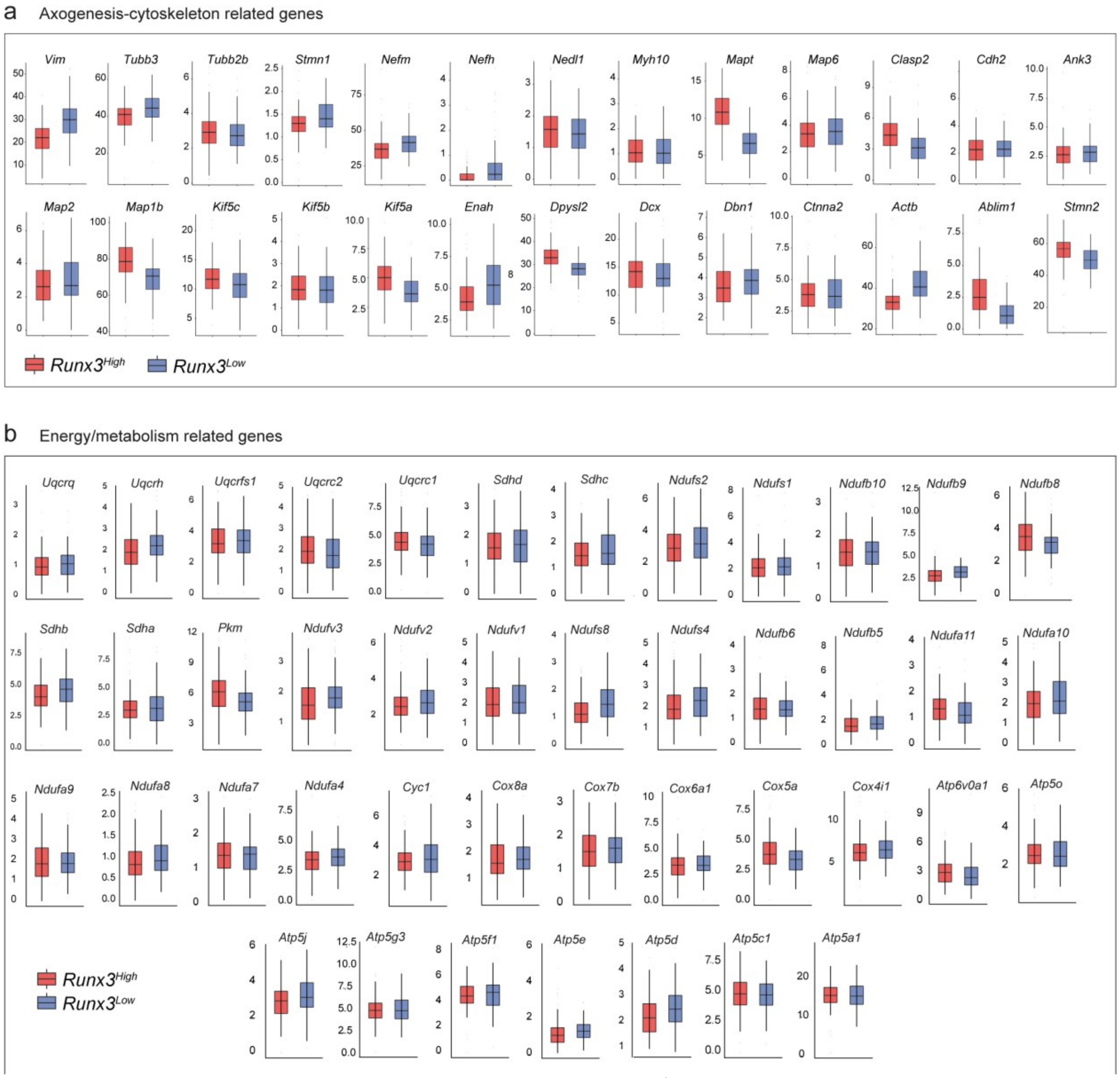

Supplementary Fig. 8. Genetic identification of *Runx3*^{High} and *Runx3*^{Low} population. a, Genes of the cytoskeleton category and involved in axogenesis. No difference observed between the two populations of E11.5 PSNs in the distribution of the most represented genes. **b**, Genes involved in energy/metabolism related functions. No difference observed between the two populations of E11.5 PSNs in the distribution of the 50 identified genes related to energy/metabolism. Boxplots visualize the summary of the data set (minimum, lower quartile, median, upper quartile, and maximum).

Ntrk3 PAN	
Probe Number	Probe Sequence
1	gggcaaagagagacatccat
2	ccgccagaaactacacttgg
3	agacgcttcccagcaagaaa
4	gacacaatttgcagggcaag
5	ggcaattgatctcagtcttg
6	taatgttgatgctggcgttc
7	gtgatattccttgagatgtc
8	ccagttctctatgtgtatgg
9	acagcattgagtgtgtgcag
10	ttgatggtcagcttctggag
11	gatgttccggagtccagaat
12	tataacgcaagtgggggttc
13	tgagccggttacttgacaag
14	gctgcagttgaagaagttct
15	acactgactgatgttcatgc
16	tcacactgatctctgggaga
17	ttctcggacagtcaggttga
18	aagtgatcacggcattgtct
19	aaaggagagccagagccatt
20	gactatccagtccacatcag
21	tggtgggtgttgatggactg
22	cattggtccagttcagattg
23	agggtcaagttgatggcatg
24	caatgcacgtcagggtgaag
25	caacactggcattgctcatg
26	ggagggtagtagacagtgag
27	actcaatgcaatgttccagg
28	aatggctgtccattgtacag
29	gtccatgtgaatgatcttgg
30	gtgggctttttgaagagcag
31	tgtagttgccattgttgtag
32	tcaggaagtggccgttgatg
33	agaaatctgtgctctctgga
34	ctcgcatcagactcaaagtc
35	tcacagtgataggaggtgta
36	aaaagtgtcttcctctggtt
37	aagtccgactgctatggaca
38	cagaaggacgcaggcaaagg
39	tcattccaaatttggaccgg

Ntrk3 FL	
Probe Number	Probe Sequence
1	cctcttgatgtgctgaacat
2	ctctcttcaacacgatgtct
3	caggaagaccttcccaaagg
4	ggcttagattgtagcactca
5	cactagcatcttgtctttgg
6	ctggaaatccttcctggcag
7	aatatgctcatgctgcaggt
8	cacacaccccatagaacttg
9	atgatgagtgggtcaccatc
10	tccatgcttcatgtattcaa
11	ccctgaggaacttgttaagg
12	cacgaggatcatggcatctg
13	agcatctgagagagccctag
14	aggctatctgactggcgatg
15	ggtgtacaaagtgctgggaa
16	actagtagattggctccaac
17	ggacatgccaaaatctccaa
18	caccctgtagtaatcagtac
19	ggtacattatgctttcaggg
20	catcactctctgtggtgaac
21	aagaataaccccgaagctcc
22	aagctggaaccatggttgct
23	attcaatgacctccgtgttg
24	aagacacggccttgggtgat
25	tttagggcagactctgggtc
26	ccagcatgacatcatacact
27	gatctccttaatattcagcc
28	ccaagaatgtccaggtagat

Supplementary Table 1. smFISH probes

Supplementary references

1. de Nooij, J.C., Doobar, S. & Jessell, T.M. Etv1 inactivation reveals proprioceptor subclasses that reflect the level of NT3 expression in muscle targets. *Neuron* **77**, 1055-1068 (2013).
2. Lallemend, F. & Ernfors, P. Molecular interactions underlying the specification of sensory neurons. *Trends Neurosci* **35**, 373-381 (2012).
3. Huang, E.J. & Reichardt, L.F. Trk receptors: roles in neuronal signal transduction. *Annu Rev Biochem* **72**, 609-642 (2003).
