# Supplementary material for: A cell fitness selection model for neuronal survival during development
Source: Nat Commun. 2019 Sep 12;10:4137. doi: 10.1038/s41467-019-12119-3 (PMC6742664; doi:10.1038/s41467-019-12119-3)
Supplement: Supplementary file 3 — Description of Additional Supplementary Files [file 41467_2019_12119_MOESM3_ESM.pdf]

Description of Additional Supplementary Files

File Name: Supplementary Data 1
Description: scRNAseq data showing gene expression in Runx3^{High} versus Runx3^{Low} subgroups of PSNs at E11.5
