# Supplementary material for: A cell fitness selection model for neuronal survival during development
Source: Nat Commun. 2019 Sep 12;10:4137. doi: 10.1038/s41467-019-12119-3 (PMC6742664; doi:10.1038/s41467-019-12119-3)
Supplement: Supplementary file 5 — Reporting Summary [file 41467_2019_12119_MOESM5_ESM.pdf]

nature research

Corresponding author(s): Hadjab and Lallemend

Last updated by author(s): 2019..07.31

Reporting Summary

Nature Research wishes to improve the reproducibility of the work that we publish. This form provides structure for consistency and transparency in reporting. For further information on Nature Research policies, see Authors & Referees and the Editorial Policy Checklist.

Statistics

For all statistical analyses, confirm that the following items are present in the figure legend, table legend, main text, or Methods section.

n/a	Confirmed	
☐	☒	The exact sample size (*n*) for each experimental group/condition, given as a discrete number and unit of measurement
☐	☒	A statement on whether measurements were taken from distinct samples or whether the same sample was measured repeatedly
☐	☒	The statistical test(s) used AND whether they are one- or two-sided<br>*Only common tests should be described solely by name; describe more complex techniques in the Methods section.*
☒	☐	A description of all covariates tested
☐	☒	A description of any assumptions or corrections, such as tests of normality and adjustment for multiple comparisons
☐	☒	A full description of the statistical parameters including central tendency (e.g. means) or other basic estimates (e.g. regression coefficient) AND variation (e.g. standard deviation) or associated estimates of uncertainty (e.g. confidence intervals)
☒	☐	For null hypothesis testing, the test statistic (e.g. *F*, *t*, *r*) with confidence intervals, effect sizes, degrees of freedom and *P* value noted<br>*Give P values as exact values whenever suitable.*
☒	☐	For Bayesian analysis, information on the choice of priors and Markov chain Monte Carlo settings
☒	☐	For hierarchical and complex designs, identification of the appropriate level for tests and full reporting of outcomes
☒	☐	Estimates of effect sizes (e.g. Cohen's *d*, Pearson's *r*), indicating how they were calculated

Our web collection on statistics for biologists contains articles on many of the points above.

Software and code

Policy information about availability of computer code

Data collection	details on data collection is given in the Methods section
Data analysis	details on the softwares used are given in the Methods section

For manuscripts utilizing custom algorithms or software that are central to the research but not yet described in published literature, software must be made available to editors/reviewers. We strongly encourage code deposition in a community repository (e.g. GitHub). See the Nature Research guidelines for submitting code & software for further information.

Data

Policy information about availability of data

All manuscripts must include a data availability statement. This statement should provide the following information, where applicable:

- Accession codes, unique identifiers, or web links for publicly available datasets
- A list of figures that have associated raw data
- A description of any restrictions on data availability

The accession number for the data used in this article is GEO (see Data Availability section), and browsing summary information for individual gene is available at https://ki.se/en/neuro/lallemend-laboratory. Data that support the findings of this study are also available from the corresponding authors upon reasonable request.

Field-specific reporting

Please select the one below that is the best fit for your research. If you are not sure, read the appropriate sections before making your selection.

☒ Life sciences ☐ Behavioural & social sciences ☐ Ecological, evolutionary & environmental sciences

For a reference copy of the document with all sections, see nature.com/documents/nr-reporting-summary-flat.pdf

Life sciences study design

All studies must disclose on these points even when the disclosure is negative.

Sample size	Sample size was determined according to our experience and literature reporting similar experiments.
Data exclusions	No sampe was excluded. Cell exclusion criteria for single cell RNAseq data are explained in the Material and Method section
Replication	All attempts at replication were successful.
Randomization	No randomization was performed since no experimental groups were used.
Blinding	No blinding was used, clustering of single cell RNAseq data was performed in an unbiased manner

Reporting for specific materials, systems and methods

We require information from authors about some types of materials, experimental systems and methods used in many studies. Here, indicate whether each material, system or method listed is relevant to your study. If you are not sure if a list item applies to your research, read the appropriate section before selecting a response.

Materials & experimental systems

n/a	Involved in the study	
☐	☒	Antibodies
☒	☐	Eukaryotic cell lines
☒	☐	Palaeontology
☐	☒	Animals and other organisms
☒	☐	Human research participants
☒	☐	Clinical data

Methods

n/a	Involved in the study	
☒	☐	ChIP-seq
☒	☐	Flow cytometry
☒	☐	MRI-based neuroimaging

Antibodies

Antibodies used	Primary antibodies used were: rabbit anti-RUNX3 (gift from Jessell TM), goat anti-TRKA (R&D Systems AF1056), goat anti-TRKB (R&D Systems AF1494), rabbit anti-TRKC (Cell Signaling 3376), mouse anti-ISL1 (Developmental Studies Hybridoma Bank 39.4D5), goat anti-RET (R&D Systems AF482), goat anti-TRKC (R&D Systems AF1404), mouse anti-βIII-tubulin (Promega G712A), rabbit anti-pAKT (Cell Signaling 4060S), chicken anti-RFP (Rockland 600-901-379S). Alexa Fluor secondary antibodies (Live Technology; 1/500)
Validation	All commercial antibody validations are available on manufacturers' websites or associated litterature

Animals and other organisms

Policy information about studies involving animals; ARRIVE guidelines recommended for reporting animal research

Laboratory animals	Wild-type C57BL6 mice were used unless specified otherwise. Runx3-/-, TrkCCreER, Raldh2-/-, Raldh1fl/fl, Raldh2fl/fl, Raldh3fl/fl and the CAGGCreERTM mouse strains have been described elsewhere (references in the text: 30,47,48,67-69). Bax-/-, R26CreERT2, TrkC-/- and Ai14 mice have been purchased from Jackson Laboratories, and NT3-/- and TrkCCre, from MMRRC. Animals of either sex were included in this study. Animals were group-housed, with food and water ad libitum, under 12hr light-dark cycle conditions.<br>Fertile white Leghorn eggs were incubated at 38ºC and embryos were staged according to Hamburger-Hamilton (HH) tables.
Wild animals	n.a.
Field-collected samples	n.a.

Ethics oversight	All animal work was performed in accordance with the national guidelines and approved by the local ethics committee of Stockholm, Stockholms Norra djurförsöksetiska nämnd.

Note that full information on the approval of the study protocol must also be provided in the manuscript.
